# Balloon-assisted sacroplasty as a successful procedure for osteoporotic sacral insufficiency fractures after failure of the conservative treatment

**DOI:** 10.1038/s41598-020-75384-z

**Published:** 2020-10-28

**Authors:** D. Schwetje, Y. El Sayed Hassan Wahd, R. Bornemann, T. R. Jansen, R. Pflugmacher, A. Kasapovic

**Affiliations:** 1 Departement of Orthopedics and Traumatology, University Hospital Bonn, Bonn, Germany; 2 Department of Orthopedics and Traumatology, Faculty of Medicine for Girls, Alzhraa University Hospital, Al-Azhar University, Cairo, Egypt

**Keywords:** Endocrinology, Endocrine system and metabolic diseases, Metabolic bone disease, Osteoporosis

## Abstract

Osteoporotic vertebral fractures without prior adequate traumatization are frequent diagnosed in orthopedics because of the increasing life expectancy and incidence of osteoporosis. The associated high mortality is caused by reduced mobilization which leads to a higher risk of infection and a bedridden state. On the other hand the diagnosis of sacral insufficiency fractures is often prolonged because of unspecific symptoms while being associated with similar risks. This article presents an overview of the present scientific literature and a retrospective analysis of patients treated via balloon-assisted sacroplasty. In 8 years, ten patients (three men and seven women) were treated. The average age was 78.4 years and the average time until the diagnosis 4.6 weeks. In most patients a significant pain reduction after the failure of conservative treatment thanks to operative treatment as well as increased mobility was observed. Only one experienced a minor surgical complication being cement leakage with nerval impaction which did not compromise her clinical outcome or satisfaction with the procedure. Balloon-assisted sacroplasty can possibly be seen as an effective symptomatic therapy in osteoporotic insufficiency fractures.

## Introduction

Sacral insufficency fractures are commonly underdiagnosed because of unspecific symptoms who are similar to lumbar spine stenosis or (pathological) lumbar spine body fractures. Besides, sacral insufficency fractures are concomitant pathologies in osteoporotic lumbar spine body fractures and therefore overseen when finding the latter^1^. Clinical symptoms are unspecific being caudal lumbar or gluteal pain, rarely sciatica or a neurological deficit^2,3^.

**Figure 1:**
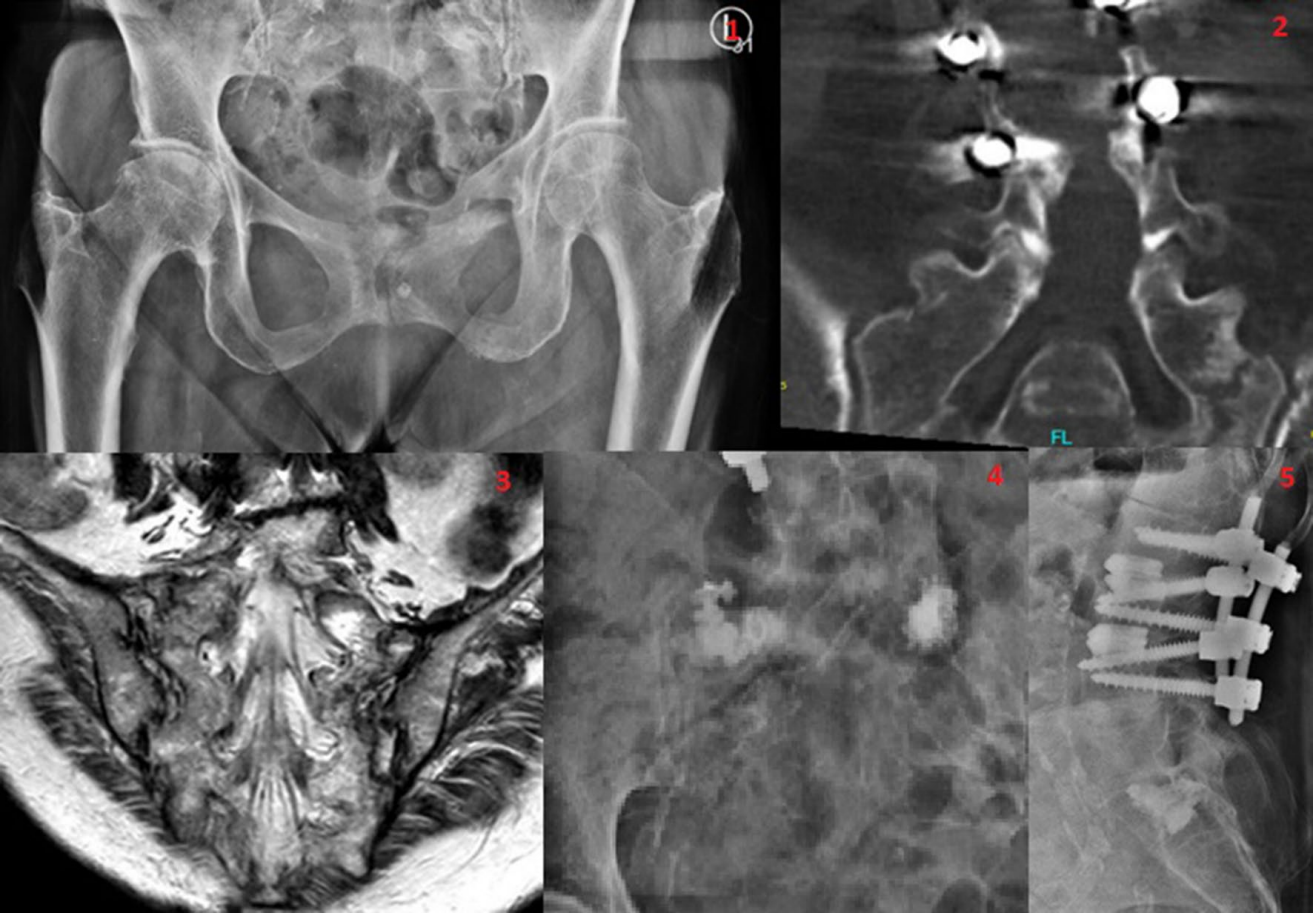
Imaginary findings of one of the patients with a bilateral sacral insufficiency fracture being the first an anterior–posterior plane of the X-ray preoperatively, the second a CT-scan in anterior–posterior plane preoperatively, the third an MRI in anterior–posterior plane preoperatively, the forth an anterior–posterior plane of the X-ray postoperatively and the fifth an lateral plane of the X-ray postoperatively.

Typically, a second look on imiginary findings in patients with persistent pain are the key to finding the right diagnosis^2^. The average time span between onset of symptoms to diagnosis is several weeks to months^1^. Explanations herefore are various. First, native X-rays cannot be viewed as the best diagnostic tool to find sacral insufficiency fractures with a sensitivity of only 20–38%^4,5^. Therefore, further radiological methods are necessary. The highest sensitivity of 96% offers scintigraphy who shows the typical H-sign (shaped as the car enterprises logo Honda)^6^. On the other hand computertomography with the reduced sensitivity of 50–75% should be combined with the elevated MRI-technology^7^. To show the difference in radiological methods in findings sacral insufficiency fractures, one patient with bilateral fractures is shown in Fig. 1.

The common Denis classification describes localisation as well as clinical symptoms. Type I is defined as fractures of the alae sacrales with seldom sciatica or neurological deficits and therefore the classical osteoporotic insufficiency fracture of the sacrum^8^.

In 40% of patients, sacral insufficiency fractures are bilateral, which is in accordance to our findings (see below)^9^.

The incidence of sacral insufficency fractures varies between different authors in current scientific papers being 1–5% in risk patients or 37/100.000 in total^10,11^. Osteoporosis is the main risk factor because the sacrum consists mainly of cancellous bone which is reduced and altered in patients with reduced bone quality^2^. Therefore, patients are typically 60–70 years old and of the female gender^5,12^. Other risk factors are Pagets disease, multiple myeloma, steroid intake and hyperparathyreodism^5,12,13^. Another reason for its frequent fracture is the mechanical function of the sacral bone. The sacrum leads vertical axial forces towards below and stabilises the pelvis against forces from below^14^.

Several authors recommend in type I fractures without neurological deficits a conservative treatment protocol consisting of initial bedrest, analgesia and physiotherapy^15^. This therapy concept carries risks such as decubitus, vein thrombosis, and pulmonary artery embolism and pneumonia in older (often multimorbid) patients^16^. One-year mortality after insufficiency fractures of the pelvis is 15% and in 20% complete reconvalescence is not achieved^14^. Since 2002, the interventional option of sacroplasty has been known, in whichthe insufficiency fracture is stabilised with bone cement similiar as in a kyphoplasty or vertebroplasty^17^. Numerous variants of the method exist, such as the percutaneous procedure with CT or radio frequency support. Their safety has been shown in numerous studies, as well as effectiveness in reducing pain and improving mobility^4,18,19^. Complications such as cement leakage and nerve injury are rare^20^.

## Methods

Over a period of 8 years (2009–2017), ten patients, including three men and seven women, were treated with a balloon-assisted sacroplasty. The average age was 78.4 years (minimum: 57 years, maximum: 88 years). The localization of the fracture was unilaterally left in 3/10 and in 7/10 on both sides. 2/10 patients were active smokers . Informed consent was drawn from all patients. All methods were carried out in accordance with relevant guidelines and regulations. The ethics committee of the university of Bonn approved this study (No. 118/19).

The ten patients were retrospectively examined and questioned for at least 2 weeks and up to a maximum of 6 years post-surgery. The Oswestry Disability Index (ODI), the Core Outcome Measures Index (COMI) and the EQ-5L of EUROQOL were used to assess clinical outcomes and therapy success. The Oswestry Disability Index (ODI) uses ten multiple-choice questions to record the percentage of physical limitations in the patient's life due to pain. The Core Outcome Measures Index (COMI) first interviews the patient with the pain intensity on the numerical pain scale and later on the extent of the physical restriction caused by the disease and the subjectively perceived quality of life of the patient. The maximum is 10, the minimum is 0. The EQ-5L was developed by EUROQOL and asks the patient's health not only for the pain but also for the restriction in everyday life, but also for the patient's mood. The evaluation is carried out using a country-specific index, which records the basic situation of the respective population, which is reflected in an index of 0.088 and Visual Analog Scale (VAS) of 0.087 according to the time trade-off method (TTO). In both instruments, a high value correlates with a subjectively perceived good health condition.

To give a better overview Table 1 contains clinical information about the patients of this study including gender, age, location, concomitant fractures, osteoporotic medication, serum vitamin D level if measured, T-Score, nicotine abuse, prior fractures, time to diagnosis, length of hospital stay and BMI.

**Table 1:** Overview of the ten patients examined in this study including gender, age, location, concomitant fractures, osteoporotic medication, serum vitamin D level if measured, T-Score, nicotine abuse, prior fractures, time to diagnosis, length of hospital stay and BMI.

Sex, age (years)	Location	Concomitant fractures	Osteoporotic medication if osteoporosis was known	Serum vitamin D level (ng/ml) [standard value: 30–100 ng/ml]	T-Score	Smoker	Prior fractures	Time to diagnosis	Length of stay, discharged to	BMI
Female 79	Bilateral	L 1,2	/	Unknown	/	Unknown	L2	1 day	12 days; geriatric ward	UNKNOWN
Female 72	bilateral	Th1, L1-2	Risedronat (orally administered), vitamine D	44.3	− 2.47	No	L 3–5	3 months	3 days, home	28.5
Female 78	Bilateral	/	Known, no therapy	38.6	Unknown	yes	/	2 weeks	21 days, home	20
Female 84	Bilateral	L4	Calcium, vitamin D	20.6	/	No	/	6 weeks	20 days, Geriatric ward	27.3
Male 57	Left	/	Calcium	23.4	− 0.9	No	/	3 months	5 days, home	35.2
Male 86	Bilateral	/	Vitamine D	28.6	− 4.1	Not active, former nicotine abusus	/	8 weeks	4 days, home	34.4
Female 88	Left	L2-3, Os pubis	Teriparatid	23.8	Unkwown	no	L1, 4–5, Th 8, 12	1 week	18 days, home	26.8
Female 87	Bilateral	/	Calcium, vitamin D	14.3	Unknown	Not active, former nicotine abusus	Th 12, L 2–3	Chronical glutealgia with acute exacerbation for several days (3–5; Patient couldn’t make a clearer observation)	12 days, Geriatric ward	23.2
Male 59	Bilateral	/	/	Unknown	/	Yes	/	1 week	21 days, home	23.8
Female 88	Bilateral	/	/	Unknown	/	No	/	Several days (3–5; Patient couldn’t make a clearer observation)	34 days, ventral cement leakage, 4/5 left foot elevater paresis; Orthopedic rehabilitation	Unknown
Female 85	Left	/	Calcium, vitamin D	Unknown	/	ja	L1	3 weeks	33 days, Geriatric ward	32.8

## Results

When asked directly whether the patients had benefited from the operation, 9/10 patients responded positively, with one patient not reporting a significant pain relief. This assessment was reflected in all questionnaires. Preoperatively, this study population showed an ODI of 73.34%, postoperatively 24.05%. In terms of COMI, there was an improvement from 8.04 to 2.62, and for the TTO from − 0.02 to 0.69. The VAS also provided postoperative relief for patients ( 0.06 to 0.57).

**Figure 2:**
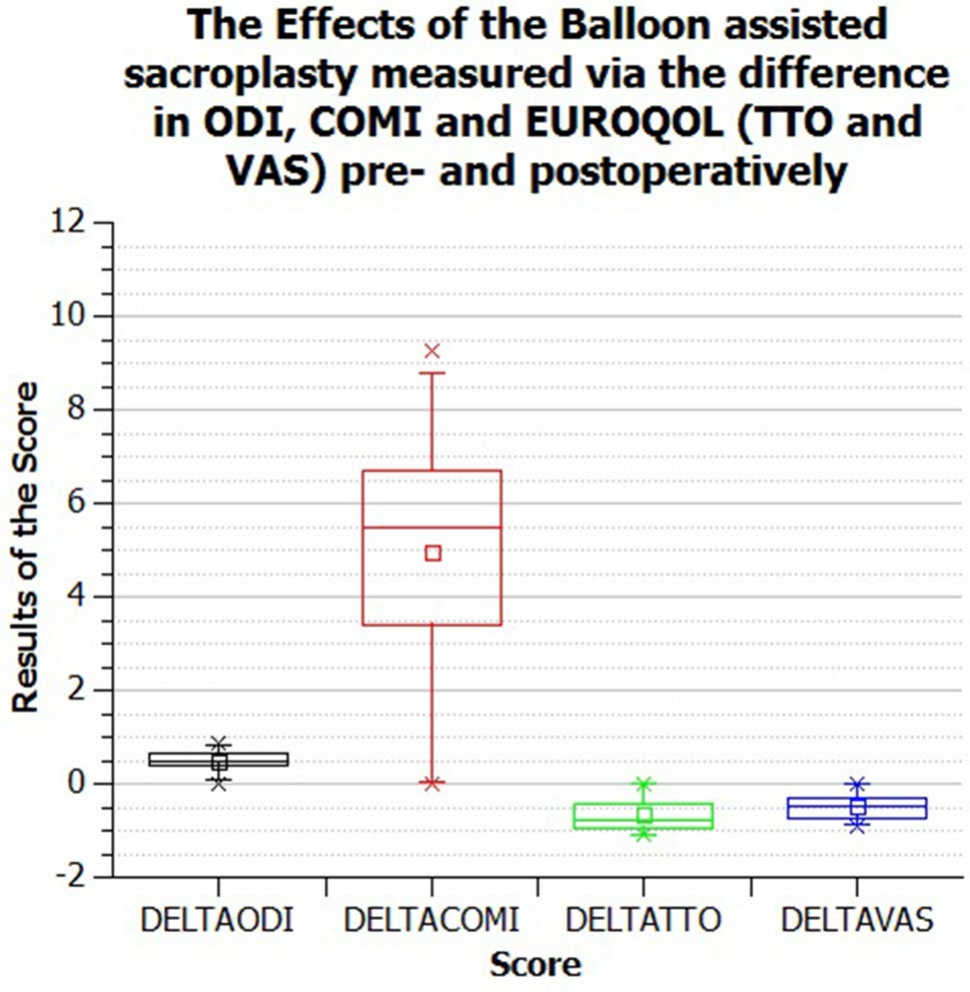
Results of the balloon-assisted sacroplasty measured by the difference of the Oswestry Disability Score (ODI), Core Outcome Measures Index (COMI) and the EQ-5L of EUROQOL with TTO and VAS, shown in the box plot.

**Figure 3:**
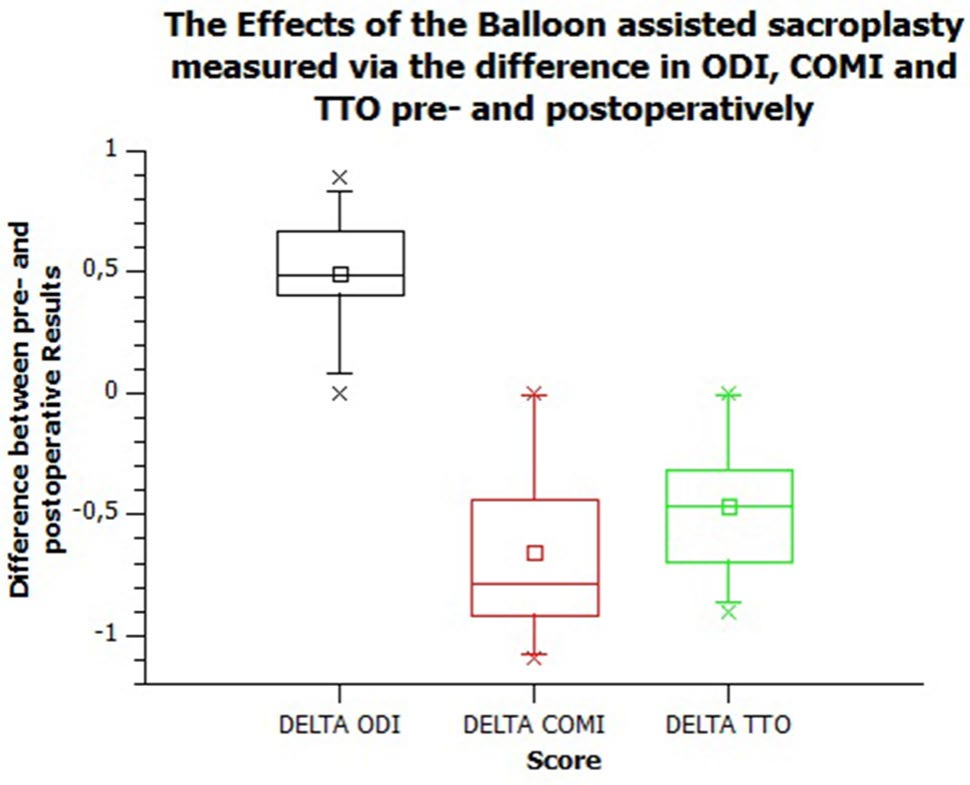
Results of the balloon-assisted sacroplasty measured by the difference of the Oswestry Disability Score (ODI), Core Outcome Measures Index (COMI) and the EQ-5L of EUROQOL with TTO, shown in the box plot.

This represents an improvement in the ODI by 49.27%, the COMI by 5.42 and the TTO by − 0.72. These results can be observed in Figs. 2 and 3 **(the latter is included to show the results of the ODI, COMI and TTO in detail better).

In the patient material presented here there was one case of cement leakage, which led to a low-grade foot lift weakness of 4/5 after Janda. The patient could be mobilized under physiotherapeutic guidance on the walker and was nevertheless satisfied with the postoperative result in pain reduction and improvement of mobility, so that she was discharged into the home environment. (ODI preoperatively: 91.11%; ODI postoperative:1.2%).

## Discussion

The sacral Insufficiency fracture is characterised by non-specific symptoms, so that the diagnosis is often prolonged. In the patients of this study, the pathology of lumbalgia and glutealgia was presented, in some as pseudoradicular femoralgias without sensomotorical deficits.

Only one patient out of ten had a fall prior to developping any symptoms. This lack of trauma in the patients ‘ history was reflected in a prolonged diagnosing period of 4.6 weeks (minimum: a few days, maximum: 3 months).

Spinal insufficency fractures are an expression of decreased bone quality in manifest osteoporosis and often the first manifestation of a metabolistic disorder. Therefore, insufficency fractures are an obvious differential diagnosis of back pain, and the sacral insufficency fracture should be considered besides a lower incidence.

It should also be pointed out that in the present study the sacral insufficiency fracture was the first symptom of osteoporosis in 2 out of 10 patients. This also demonstrates the need to consider the sacral fracture as a differential diagnosis for a sudden lower back pain.

At the same time, osteoporosis was known in 8 out of 10 patients (manifest in 4 out of 10), but only 2 out of 10 received a specific, antiresorptive therapy in addition to the general supplementation of vitamin D and calcium.

The fact that the diagnosis and therapy of osteoporosis in this group of patients can be described as deficient is also shown in the perioperatively determined vitamin D content of 29.88 ng/ml (3 patients not tested) (minimum 14.3 ng/ml; maximum 44.3 ng/ml) [standard value: 30–100 ng/ml]. **While the so-called german health check screens a comprehensive diagnosis of cardiovascular and metabolic diseases such as diabetes mellitus, osteoporosis often fades into the background. However, if one considers the mortality, which is increased by decreasing mobility and associated risk of infection, the lack of or delayed correct diagnosis and thus increased treatment and care costs is a loss business.

Breuil et al. had similar results in their clinical studies: 53.8% of patients with an osteoporotic sacral fracture were known to have osteoporosis (57.6% manifest), but only 30.9% of patients with known osteoporosis received antiresorptive therapy. High rates of vitamin D deficiency (n = 27/31), secondary hyperparathyroidism (n = 16/31), and hypocalcemia (n = 14/49) were also found.

The DEXA measurement was able to show osteoporosis (T score <  − 2.5) in 12 of the 19 patients tested^21^. These figures are in line with the results presented here. Diagnosis and therapy of osteoporosis continue to be a challenge in today's medicine. This obvious gap in care should be closed at last by the surgeon when an osteoporotic (sacral) fracture is diagnosed and a guideline-based therapy should be started^22^.

Sacroplasty is considered a second-line procedure if conservative therapy has failed^8^. In addition to anesthesiological problems in the elderly and often multimorbid patients, critics mention the surgical complications. In particular, cement leakage with the risk of postoperative nerval lesion is to be mentioned. Numerous authors have demonstrated the low perioperative risk (especially with CT control) and the often asymptomatic perineural cement leakage^9,23^. In the patient material presented here there was one case of cement leakage, which led to a low-grade foot lift weakness of 4/5 after Janda. The patient could be mobilized under physiotherapeutic guidance on the walker and was nevertheless satisfied with the postoperative result in pain reduction and improvement of mobility, so that she was discharged into the home environment. (ODI preoperatively: 91.11%; ODI postoperative:1.2%).

After 17.1 days in average, the ten patients were discharged significantly improved in regard to former pain level and immobility. Most patients (6/10) could be discharged home. If these results are compared with those of the conservative ones, the latter shows a longer period of time until convalescence. The healing time of the conservatively treated sacrum insufficiency fracture according top the scientific literature is 1 year^24^.

It includes bed rest, analgesic and physiotherapy. The risks of immobilization have already been outlined in the introduction. Patients in this study had previously been treated conservatively except for one patient, but not improved, so the indication was given to the surgical procedure. There are numerous options for surgical care of the sacral insufficiency fracture, the balloon-assisted sacroplasty is one of those^19^. Guidelines on which procedure to choose do not exist (yet).

Further aspects can be observed in the more detailed analysis of the success of sacroplasty in terms of pain reduction and improvement of quality of life. Contrary to expectations, there is no negative correlation between diagnosis time and postoperative ODI.

This is similar to the results of EuroQol. A long period of time from the onset of symptoms to diagnosis (and surgical therapy) does not seem to have a negative influence on the convalescence period. Almost all patients were preoperatively mobile with a walker, so that reduced mobility as an outcome parameter can also not be proven.

Also, the average weight, which is to be classified as overweight, has already been mentioned. In this aspect, there was also no clear trend between the individual patients in terms of a positive factor for the outcome having normal weight. Consequently, it is not possible to deduce a clear risk factor of prolonged convalescence after balloon-assisted sacroplasty, also due to the small group of patients.

## Conclusions

Sacral insufficiency fractures are commonly under- or late diagnosed because other diseases such as osteoporotic lumbar body fractures or lumbar spine stenosis with similar clinical symptoms. This article showed standard conservative therapy consisting of initial bedrest, early onset physiotherapy and analgesia. In cases of its failure, minimally invasive operative procedures are indicated. Balloon-assisted sacroplasty seems to be an effective symptomatic therapy in osteoporotic insufficiency fractures. In most patients a significant pain reduction after failure of conservative treatment thanks to operative treatment as well as increased mobility was observed. With only one surgical complication being ventral cement leakage resulting in a 4/5 foot elevator paresis without need of a re-intervention, this surgical treatment may be considered a safe treatment option when conservative treatment has failed or to increase recovery time of the patients.
